# Bioactive Supramolecular Polymers for Skin Regeneration Following Burn Injury

**DOI:** 10.1021/acs.biomac.5c01107

**Published:** 2025-07-16

**Authors:** Penelope E. Jankoski, Abdul-Razak Masoud, Jenna Dennis, Sophia Trinh, Loria R. DiMartino, Jessica Shrestha, Luis Marrero, Jeffery Hobden, Jeffrey Carter, Jonathan Schoen, Herbert Phelan, Alison A. Smith, Tristan D. Clemons

**Affiliations:** 1 School of Polymer Science and Engineering, 5104 University of Southern Mississippi, Hattiesburg, Mississippi 39406, United States; 2 12258 Louisiana State University Health Sciences Center, New Orleans, Louisiana 70112, United States; 3 University Medical Center, New Orleans, Louisiana 70112, United States; 4 Center for Molecular and Cellular Biosciences, 5104 University of Southern Mississippi, Hattiesburg, Mississippi 39406, United States

## Abstract

Severe deep dermal burns present a significant challenge for the clinician, often resulting in complications including infection, scarring, and potentially multisystem organ failure. The current standard of care, which involves debridement and skin coverage, has improved survival rates but remains insufficient for optimal tissue regeneration and functional recovery. Additionally, there can be limited donor skin availability with severe burns, leading to the use of skin substitutes to be applied with varying degrees of success reported. Biomaterial scaffolds, designed to reduce the reliance on skin grafting, could promote improved healing and patient outcomes. Recent research has focused on promoting the proliferative phase of wound healing through the use of extracellular matrix (ECM) mimetic scaffolds; however, these constructs continue to exhibit critical limitations, including mechanical fragility, heightened infection susceptibility, limited morphological conformity to host tissue architecture, and the necessity for secondary surgical intervention for scaffold retrieval. This study presents a bioactive supramolecular polymer capable of rapid self-assembly into nanofibers, which act as a scaffold to promote tissue regeneration following burn injury. The scaffold is biocompatible, biodegradable, and capable of presenting a bioactive peptide designed to reduce acute inflammation and promote keratinocyte migration in the scaffold. The supramolecular polymers significantly accelerated early wound healing in a clinically relevant deep dermal murine burn injury model. This work provides a promising approach to the development of biomaterials that combine both therapeutic strategies, with scaffolding to promote skin regeneration following severe burn injury.

## Graphical abstract

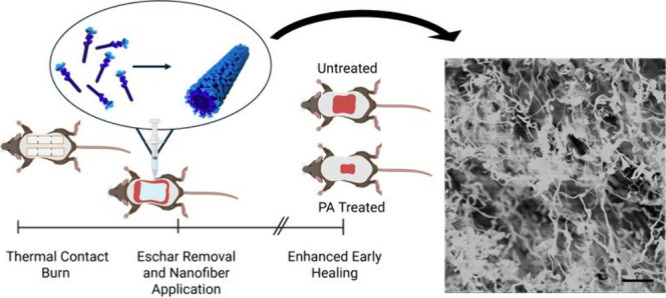

## Introduction

Skin is the largest organ in the human body serving as the primary barrier of the immune system, protecting internal organs from environmental factors such as pathogens, UV radiation, and physical trauma. It provides the first line of defense against exposure to the outside world.^1,2^ Beyond acting as a barrier, the skin contains many components that contribute to its myriad of functions including the ability to modulate body temperature, aid in the synthesis of vitamin D, facilitate sensory perception, and many others.^1,3^ Thus, damage to this vital organ presents a unique threat not just to the injured skin but to the body’s ability to maintain homeostasis.^4^

Severe burns, which affect not only the epidermis but also the deeper dermal layer of the skin, can result in significant morbidity and even mortality.^5,6^ According to the World Health Organization (WHO), an estimated 180,000 deaths occur annually worldwide due to burns, with over 11 million people requiring medical treatment for burn injuries each year.^7,8^ These injuries typically result from thermal, chemical, or electrical sources, leading to extensive tissue damage that disrupts both the structural and functional integrity of the skin.^9^ Approximately 90% of burns requiring medical intervention arise from a thermal source (scalds, dry heat, or contact injuries) and produce wounds that are topographically complex, as different regions of the skin are often exposed to varying intensities of the insult.^10^

Deep dermal burns pose a significant threat, where damage to the epidermis, dermis, and skin structures like sweat glands, hair follicles, and collagen fibers severely impacts skin functionality.^11,12^ Beyond the immediate threat to life, severe burns can lead to numerous complications, including infections, hypovolemic shock, and long-term metabolic alterations.^1,13^ The loss of skin’s protective functions necessitates comprehensive medical interventions to address both acute and chronic health concerns. The treatment of these burns depends on a series of compounding factors, from the nature of the burn, size, depth, and location, to patient health and the resources of the surgical team administering treatment.^14,15^ The current standard of care involves debridement and wound cleaning, adequate resuscitation, and finally skin coverage of the affected area.^16,17^ Burn patients are at an elevated risk of infection, commonly from drug-resistant microorganisms, leading to longer hospital stays, delayed healing, scarring, and higher mortality.^18,19^ Prolonged healing times in deep dermal burns significantly increase the risk of impaired tissue regeneration and long-term scarring, both of which remain major limitations of current therapeutic strategies.^8,20^ These outcomes not only pose persistent clinical challenges but also lead to substantial reductions in patient quality of life, underscoring the urgent need for innovative approaches that accelerate wound closure and promote functional, scar-minimizing tissue regeneration. This has been an area of focus as it has been established that accelerated healing reduces challenges in the inflammatory phase and importantly reduces overall risks of infection.^21–24^

Despite the significant social and financial burden of healing burn wounds, current treatment options are suboptimal.^5^ Numerous regenerative scaffolds and skin substitutes have been proposed for the management of skin injuries following burns, including both biological (autologous, allogeneic, and xenogeneic) and synthetic (biodegradable and nonbiodegradable) regeneration scaffolds.^11^ Some of the deficiencies of current therapeutic interventions include fragility of the scaffold, increased risk of infection, graft failure, prolonged processing times, the need for secondary surgical interventions, and high costs.^1,25^ Autologous skin grafts rely on the patient having skin available to harvest and the patient’s overall health status. While synthetic alternatives are readily available, there remain challenges in their application. For example, Matriderm is a highly porous membrane composed of type I collagen and elastin, frequently and effectively used in combination with split-thickness skin grafting to facilitate dermal regeneration in burns and chronic wounds. However, its application necessitates the use of a significant amount of patient-derived skin.^26,27^ Alternatively, Integra is a biocompatible hydrogel sheet composed of a porous matrix made from cross-linked bovine tendon collagen and chondroitin-6-sulfate derived from shark fins. This structure is topped with a semipermeable polysiloxane layer, which helps preserve its integrity.^28^ However, Integra is not biodegradable, requiring two surgical procedures for full effectiveness, and it carries a risk of infection beneath the silicone layer, which may detach over time.^29^ These scaffolds can significantly aid wound management, however, not without their challenges. As a result, there remains a continued need for functional, safe, easy to apply scaffolds to promote skin regeneration following burn injury.

Peptide amphiphiles (PAs) are an exciting class of supramolecular polymers that are composed of lipids and amino acids, highly suitable for tissue regeneration applications. They self-assemble through noncovalent interactions, achieving a thermodynamic equilibrium between the formation of a hydrophobic core due to the collapse of the lipid components upon solvation, and intermolecular hydrogen bonding between the amino acid residues.^30^ This produces nanostructures, which can lead to fiber formation through careful selection of amino acid residues that engage in strong hydrogen bonding driving cohesive forces between monomer units and excluding water, creating tightly packed fibrils.^31,32^ One-dimensional nanofibers form an entangled three-dimensional network that closely mimics the architecture of the extracellular matrix (ECM), making them ideal candidates for tissue scaffolding. These supramolecular polymers—composed of monomers held together by noncovalent interactions—can withstand high shear forces, such as those encountered during needle-based delivery, and rapidly reassemble into ECM-analogous scaffolds, providing a promising platform for regenerative medicine applications.^33,34^ Peptide-based biomaterials are attractive for regenerative medicine applications as signaling in the ECM is primarily mediated by proteins whose function can be mimicked with short peptide epitopes.^35^ Furthermore, in the design of these materials, a bioactive epitope can be included to enhance tissue regeneration and cell migration.^34,36,37^

As an example, work from Jarrahy et al. applied fiber-forming peptides that express the RGDS peptide epitope, to promote fibroblast proliferation and wound re-epithelialization in a partial thickness burn injury model.^38^ This work established a basis for utilizing a functionalized scaffold that acts as the matrix and therapeutic in the treatment of thermal skin injuries. Motivated by current challenges in the treatment of complex topography deep dermal wounds, we aimed to develop a tunable platform that could increase the rate of wound healing and skin regeneration following major burn injury. Furthermore, we looked to apply our materials to a clinically relevant murine model that reflects the complexities of deep dermal wound care to validate their translational potential.

## Materials and Methods

### Materials

Rink amide polystyrene resin, Fmoc-protected amino acids, and ethyl cyanoglyoxlate-2oxime (Oxyma) were purchased from CEM peptides. Dichloromethane (DCM), diethyl ether, trifluoroacetic acid (TFA), *N*-dimethylformamide (DMF), acetonitrile, diisopropylcarbodiimide (DIC), triisopropylsilane (TIS), ethane-1,2-dithiol (EDT), uranyl acetate, and all other solvents were purchased from Thermo Fisher Scientific (USA) or Sigma-Aldrich Corporation (USA) at the highest purity. Dulbecco’s modified Eagle medium (DMEM), heat-inactivated fetal bovine serum (FBS), and penicillin–streptomycin were all purchased from Thermo Fisher. Sterile, tissue-treated 96-well plates were obtained from CELLTREAT (USA). The CyQUANT Lactate Dehydrogenase (LDH) Assay and the Live/Dead Imaging kit was purchased from Thermo Fisher (USA). Hexafluoroisopropanol (HFIP) was obtained from AA Blocks, and Nile Red was purchased from APExBIO. Isoflurane USP was purchased from Covetrus (batch no. G48D23A) (USA). Meloxicam ER (lot no. 222-05468474) and buprenorphine HCl in polymer (lot no. 222-05509534) were purchased from Wedgewood Pharmacy (USA). Mason’s Trichrome staining kit was purchased from StatLab (SKU no.: KTMTR2LT) (USA).

### Peptide Amphiphile Synthesis

Peptide amphiphiles were synthesized on a Liberty Blue 2.0 automated peptide synthesizer (CEM) through standard 9-fluorenyl methoxycarbonyl (Fmoc)-based solid phase peptide synthesis. Peptide synthesis was performed at 0.25−1 mmol scale using Rink Amide Polystyrene Resin (0.3 mmol/g loading, 100–200 mesh). Deprotection of Fmoc-protecting groups was carried out using 20 v/v% piperidine in DMF. Each amino acid addition was carried out using Fmoc-protected amino acids (0.2 M), DIC (1M), and Oxyma (1 M) in DMF. After the final Fmoc deprotection, the resin beads were washed 3× using DCM. The peptide then underwent global deprotection and cleavage from the resin beads through gentle shaking in TFA/TIS/H_2_O/EDT (95:2.5:2.5:2.5) cleavage cocktail for 3 h at room temperature. Peptide amphiphiles were then precipitated in cold diethyl ether and collected via centrifugation. The peptide pellet was then resuspended in diethyl ether and chilled for 4 h. It was recentrifuged, and the diethyl ether supernatant was decanted from the peptide pellet, which in turn was allowed to air-dry. Crude peptides were purified on a Prodigy preparative reverse-phase HPLC (CEM) with a water/acetonitrile gradient (containing 0.1% NH_4_OH). The mass and identity of the eluting fractions containing the desired peptides were confirmed using electrospray ionization (ESI)–mass spectrometry (MS) on a Thermo Scientific Orbitrap Exploris 240. Purity was confirmed using liquid chromatography mass spectrometry, with a demonstrated purity of greater than 95%. Initial targets for this work were synthesized at the NSF-supported BioPACIFIC MIP facilities following similar protocols as above utilizing a Gyros Protein Technologies Symphony X Peptide Synthesizer.

#### Diluent PA Synthesis

The following peptide sequence C_16_V_3_A_3_E_3_ was synthesized on Rink Amide MBHA resin making use of the CEM Liberty microwave-assisted peptide synthesizer and protocols described above.

#### REGRT PA Synthesis

The following peptide sequence C_16_V_3_A_3_E_3_G_4_REGRT was synthesized on Rink Amide MBHA resin, making use of the CEM Liberty microwave-assisted peptide synthesizer and protocols described above.

### Peptide Amphiphile Nanofiber Preparation

The resulting powders were dissolved in 100 mM HCl and lyophilized to neutralize any lingering TFA. The PAs were then weighed out into Eppendorf tubes to be at 10 mM concentration with a working volume of 1 mL. The PAs were then dissolved in Milli-Q water, and pH was slowly adjusted to a pH between 7 and 8 using 1 M NaOH being careful not to overshoot. The PAs were then lyophilized and resuspended in working volume to obtain 10 mM stock solutions. To prepare the 20% bioactive PA, 200 μL of 10 mM REGRT PA and 800 μL of 10 mM diluent PA were mixed. The bioactive PA was then lyophilized. The dry powders were dissolved in HFIP and left to evaporate overnight. They were then redissolved in 1 mL of DI water and lyophilized. Lyophilized powders were then stored at −20 °C until use.

### Peptide Amphiphile Nanofiber Preparation for Animal Studies

PAs for application to animals were dissolved in 990 μL of sterile Ringer’s lactate solution. These PAs were thermally annealed at 80 °C for 30 min and allowed to slowly cool back to room temperature. For animal studies, 1 M CaCl_2_ was prepared in DI water and sterilized using the liquid setting on the autoclave. Following annealing, 10 μL of CaCl_2_ was added to promote gelation through ionic cross-linking. Sterile Ringer’s lactate solution was prepared with an equivalent amount of CaCl_2_ to act as a negative control.

### Liquid Chromatography Mass Spectrometry (LC-MS)

The purity of PA molecules was confirmed using liquid chromatograph–mass spectroscopy (LC-MS), which was performed using an Agilent 1200 system with a Phenomenex Gemini C18 column (100 × 1.00 mm; 5 μm) for basic conditions. The mass detector (MS) was an Agilent 6520 Q-TOF MS. All gradient methods followed: acetonitrile at 5% for 5 min at 50 μL/min, 5–95% over 25 min at 50 μL/min followed by 95% for 5 min at 50 μL/min. Ammonium hydroxide (0.1% v/v) for basic conditions was added to all solvents. Peaks were detected at λ = 220 nm.

### Nile Red Assay

Stock solutions of Nile Red were prepared at 10 mM in DMSO and subsequently diluted with deionized (DI) water to achieve a final working concentration of 100 μM. PA samples were diluted in DI water to produce a concentration range of 0 to 500 μM. In a 96-well plate, 90 μL of PA solution and 10 μL of Nile Red solution were added to each well, mixed thoroughly, and incubated at room temperature for 3 h, with intermittent tapping to promote incorporation. Each condition was performed in triplicate. After incubation, samples were analyzed using a BioTek Synergy H1 Microplate Reader (Agilent), with excitation set to 550 nm and emission measured in 2 nm increments from 580 to 720 nm. The mean maximum relative fluorescence units (RFU) were plotted against the logarithm of the concentration, and the critical aggregation concentration (CAC) was determined as the intersection point of the curves corresponding to the absence and presence of fluorescence.

### Circular Dichroism (CD)

PAs were prepared as described above and diluted to 100–500 μM in Milli-Q water. CD spectra were recorded in a 1 mm-path-length cuvette on a J-815 (JASCO, Easton, Maryland) spectropolarimeter. Continuous scanning mode was used with a scanning speed of 100 nm per minute over a measurement range of 190–300 nm. The high-tension voltage (HT) was also monitored to ensure that the measurement was not saturated. Three measurements were obtained, and the buffer sample was run as a background that was subtracted.

### Transmission Electron Microscopy (TEM)

200-mesh copper grids (Ted Pella) were used as purchased. PA solution was diluted to 0.5 mM, and 5 μL was dropped on the grid and left to sit for 5 min. Excess solution was wicked away, and a drop of uranyl acetate was added as a stain to sit for 2 min. Excess solution was wicked away, and 5 μL of deionized water was added and left to sit for 5 min. The solution was wicked away, and grids were left to air-dry prior to visualization using a JEOL JEM120i transmission electron microscope.

### Scanning Electron Microscopy (SEM)

Stainless steel stubs were prepped with adhesive carbon black conductive tape. PA solutions were diluted to 2 mM and applied dropwise. Samples were then left to sit for 5 min and lyophilized using a Benchtop Pro Lyophilizer (SP Scientific). Samples remained under vacuum until they were run on SEM to prevent absorption of water as they are hygroscopic. Samples were run using a Zeiss Sigma VP field-emission SEM in a N_2_ environment.

### Rheology

Rheological measurements were conducted using a strain-controlled ARES rheometer (TA Instruments) fitted with 25 mm cone and plate steel geometry, maintaining a gap height of 0.3 ± 0.05 mm. To establish the viscosity profile of the material, a steady-rate sweep test was performed across a shear rate range of 0.01 to 10 s^–1^. 450 μL of 10 mM PA in DI water prepared from lyophilized stocks was placed on the steel plate, followed by an equal volume of 10 mM CaCl_2_ solution pipetted on top. The samples were allowed to gel for 15 min, after which 450 μL of water was carefully removed, and the test was performed. Measurements about the storage and loss modulus of the samples were made using dynamic motor mode, with cross-linked 10 mM PA samples as described above. To determine the linear viscoelastic regime, a dynamic strain sweep was performed, holding the frequency constant at 1 Hz. The PAs were investigated using a dynamic strain sweep from 0.01 to 100% strain. Then, a frequency sweep from 1 to 100 rad/s was performed at a fixed strain of 1%, to determine the material response within the LVR where solid-like behavior dominates.

### Cell Work

#### Cell Culture Maintenance

Human embryonic kidney cells (HEK 293) were maintained in Dulbecco’s modified eagle medium, supplemented with 10% fetal bovine serum (FBS) and 0.1% penicillin–streptomycin. Cells were cultured at 37 °C, 5% CO_2_ in tissue-treated flasks and used at confluence of ∼80%.

#### Cytotoxicity

HEK293 cells were seeded in a 96-well plate (1 × 10^5^ cells/mL, 100 μL volume per well). Seeded cells were incubated at 37 °C and 5% CO_2_ overnight to allow cells to adhere. Following adherence, PA solution was added to the media to incubate, and each concentration was performed in triplicate. Nuclease-free water was used as a spontaneous control, and Triton X-100 was used as a positive control for 100% cytotoxicity (i.e., complete LDH release). Plates were incubated for 24 h at 37 °C and 5% CO_2_, before collecting media to assess LDH release with the CyQUANT LDH assay following the manufacturer's protocols. A microplate reader was used to assess the absorbance at 490 nm with a reference wavelength of 690 nm. % Cytotoxicity was calculated using eqs 1 and 2 below.

$$LDH\;Activity=Absorbance_{sample,490}-Absorbance_{sample,680}$$ 1

$$\%Cytotoxicity=\left(\frac{LDHActivity_{sample}-LDHActivity_{spontaneous}}{LDHActivity_{Max\;Lysis}-LDHActivity_{spontaneous}}\right)\times 100$$ 2

Above 2 mM, there are significant challenges from the peptide solution being gelled by ions in DMEM, producing nonhomogeneous distributions of PA and cells. In order to obtain LDH data for these higher concentrations, and of the gelled systems as a whole, PAs were gelled within the well by adding 50 μL of sample and 50 μL of 10 mM CaCl_2_ solution. PAs were left to gel for 15 min, and 50 μL of excess liquid was pipetted off leaving only gelled PA behind. Cells were then gently plated on top of the gelled layer at 1 × 10^5^ cells/mL, 100 μL volume per well, and left to incubate overnight. LDH was then performed as described above using supernatant from each well.

#### Live Well Confocal Imaging

To support the LDH assay results and visualize cells within the gels, samples were prepared as described for gelled cytotoxicity. Cells were plated in a glass-bottom 96-well plate (1.5 glass), and after 24 h of coincubation, 70 μL of supernatant was removed from each well and 80 μL of live stain was added. In this study, Thermo Fisher Live/Dead Cell Imaging Kit was used; however, BOBO-3, the dead cell stain, complexes with PAs, making visualization of dead cells impossible. Cells were incubated at room temperature for 15 min before imaging on a Leica STELLARIS STED Super-Resolution Confocal Microscope. Z-stacks were taken at each concentration to visualize cells migrating within the gels.

### *In Vivo* Studies

#### Burn Wound Model

Animal experiments were conducted in accordance with protocols approved by the Institutional Animal Care and Use Committee (IACUC) at Louisiana State University Health Sciences Center – New Orleans (Protocol #2403). A total of 36 male C57BL/6J mice (JAX #000664) were procured from Jackson Laboratory (Bar Harbor, Maine, USA) for use in this study. Mice were housed under standard conditions (20–26 °C, 12 h light/dark cycle) and acclimated for 1 week prior to experimentation. On the day of the experiment, anesthesia was induced using 3% isoflurane in oxygen. The dorsal surface of each mouse was shaved, and analgesia was administered via subcutaneous injection of buprenorphine SR (0.01 mg/kg) and meloxicam SR (5 mg/kg) into the right and left groin regions. Six square full-thickness scald wounds (1 cm × 1 cm) were created on the dorsum using a heated billet applied for 10 s. The following day, the formed eschar was surgically removed, and treatment was applied. The burn and subsequent eschar removal generated a wound that was visibly through all layers of the skin, and deemed a full-thickness injury. Mice were randomly assigned to one of three treatment groups: (1) Ringer’s solution supplemented with CaCl_2_, (2) diluent PA nanofibers, or (3) bioactive RG PA nanofibers. The Ringer’s solution (supplemented with calcium) was a solution of Ringer’s lactate, which had been enhanced with an addition of CaCl_2_ to mimic the addition of calcium chloride present to promote ionic gelation within the PAs, resulting in a Ringer’s lactate with 10 mM additional calcium. PAs were prepared as described above, resulting in final concentrations of 10 mM PA and 10 mM CaCl_2_, which was found to be stiff enough to remain where placed, but capable of injection through the syringe. Approximately 400 μL of treatment were placed on each wound, providing good coverage of the wound area. Wounds were covered with transparent dressings (Tegaderm) and secured with bandages (Coban). Wound progression was monitored and photographed at defined time points (days 3, 7, 11, and 15) following treatment. Pain management was provided as needed based on animal monitoring. At the end point, mice were euthanized with 5% isoflurane in oxygen over a 40–60 min period. Wounds along with surrounding skin tissue were harvested and processed for histological analysis.

#### Kinetics of Healing

Images were taken at day 0 (burn), day 1 (eschar removal and treatment), day 3, day 7, day 11, and day 15 time points and analyzed using ImageJ to monitor wound closure over time via digital planimetry. Each picture contained a ruler, which was used to set the scale of pixels to cm^2^ to allow for comparison across photographs. Images were analyzed by a single individual to prevent bias and to ensure all images were treated the same. Wound closure was calculated using eq 1 below. Data was then analyzed using GraphPad Prism statistical software (Version 9, Dotmatics, California, USA) for a one-way analysis of variance (ANOVA) with a posthoc Tukey test (α = 0.05) for means comparison within each group.

$$\%Wound\;Closure=\left(1-\frac{Area\;of\;Wound_{Day,n}}{Area\;of\;Wound_{Day,1}}\right)\times 100$$

#### Histological Evaluation

Histological evaluation of skin tissue sections was performed using Masson’s Trichrome staining, following the manufacturer’s protocol. At the time of euthanasia, excised wound tissues were immediately fixed in zinc-buffered formalin, with wound margins marked using India ink. 5 μm-thick sections were prepared using a Thermo Scientific HM325 microtome. Sections were subsequently stained with Masson’s Trichrome, dehydrated, cleared, and mounted with coverslips. Tissue morphology was examined under a Nikon E300 light microscope equipped with a 4× objective (NA 0.10) and an Olympus DP23 camera. Images of 10 fields were taken for each slide. Histological assessment was performed using an established scoring system for murine cutaneous burn wounds, and analyses were conducted using Olympus CellSens software.^39^

## Results and Discussion

### Peptide Amphiphile Nanofiber Characterization

Two PA monomers were synthesized for this study, designed to produce a supramolecular polymer scaffold mimetic of the natural ECM (Diluent PA) and to include bioactivity to promote keratinocyte migration and reduce early-stage inflammation (RG PA). The diluent PA consists of a hydrophobic palmitic acid tail coupled to three alanine and three valine residues to enhance intermolecular hydrogen bonding and facilitate monomer cohesion within the nanofibers, and three glutamic acids to impart amphiphilicity and aqueous solubility (Figure 1A). The REGRT PA maintains the same sequence of the diluent PA, with an additional bioactive peptide sequence, REGRT coupled to four glycines, as a spacer to help promote the external presentation of the bioactive peptide from the nanofiber surface (Figure 1B). The peptide REGRT has demonstrated promise in skin regeneration for its ability to reduce early inflammation following injury, resulting in accelerated kinetics of healing.^40,41^ The sequence acts by activating the extracellular signal-regulated kinase (ERK) pathway which aids in the regulation of cellular growth, proliferation, differentiation, and response to stress, making it incredibly important in the wound healing cascade.^42,43^ REGRT was chosen over other commonly used epitopes for wound healing based on this demonstrated specificity in activating the ERK pathway. Other epitopes such as RGDS, although supportive of wound healing, have been known to produce nonspecific interactions through increased cell adhesion and migration;^44,45^ hence, the use of REGRT provided the opportunity to probe the impact of the bioactive scaffold on timing of the wound healing cascade directly. The ERK pathway can also be affected by external stimuli, like oxidative stress, and can modulate immune cell activation, further providing an opportunity to impact acute and chronic inflammation.^46^ We hypothesized that by tethering this peptide sequence to a PA nanofiber, it would localize this bioactive sequence at the site of injury and improve its bioavailability, while maintaining its ability to activate the ERK pathway.

**Figure 1:**
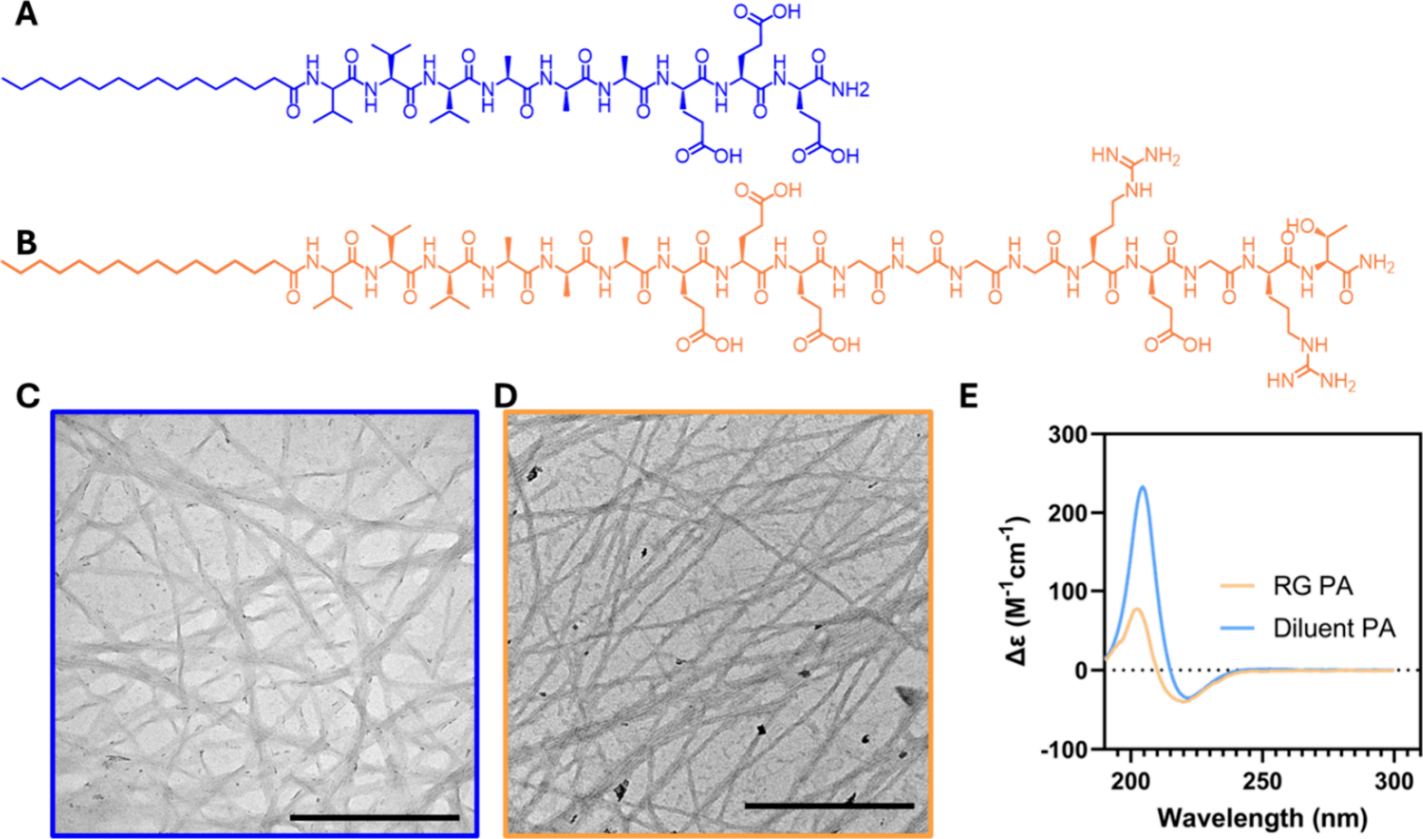
Characterization of the supramolecular polymerization of the bioactive PA nanofibers. Molecular structures of (A) the diluent PA and (B) the REGRT PA. Transmission electron microscopy (TEM) of (C) the diluent PA nanofibers and (D) the RG PA nanofibers. TEM scale bars are 500 nm. (E) Circular dichroism spectra of the RG PA nanofibers (orange) and the diluent PA nanofibers (blue).

By constructing the REGRT PA on the same backbone as the diluent PA, we can coassemble the two monomers to form supramolecular nanofibers with specific densities of the bioactive sequence expressed. In this study, the bioactive PA was used exclusively at a concentration of 20 mol % bioactive and 80 mol % diluent PA, with this coassembly referred to as the RG PA nanofibers. The decision to utilize the bioactive sequence at 20 mol % originated from concerns with the highly cationic nature of the REGRT motif being presented from the nanofiber, as it could penetrate the cellular plasma membrane, causing membrane disruption and potential toxicity.^47,48^ Furthermore, supporting literature with similar molecules has established that bioactivity is maintained at similar incorporation percentages of therapeutic pro-regenerative peptide epitopes.^38,49^ The PA monomers were synthesized by automated solid phase peptide synthesis and purity confirmed by high-resolution liquid chromatography–mass spectrometry (LC-MS, Supporting Information Figure S1 and S2). A Nile Red assay was used to determine the critical aggregation concentration (CAC) of the PA monomers, where Nile Red is a solvatochromic dye that produces intense fluorescence when in hydrophobic environments.^32,33^ Below the CAC, PA molecules cannot self-assemble, and no fluorescence is observed. As the concentration of PA molecules increases, nanofibers begin to form, producing the hydrophobic core of the structures. The Nile Red can then reside in this hydrophobic environment, and in turn, the resulting increase in fluorescence can be monitored to assess the onset of nanofiber formation (i.e., the CAC). A CAC of approximately 6 μM was observed for the diluent PA, while a CAC of approximately 4 μM was observed for the RG PA nanofiber (Figure S3). This result suggests that the inclusion of 20 mol % of the REGRT PA into the coassembly did not significantly alter the concentration required for the onset of PA supramolecular polymerization to occur.

Transmission electron microscopy (TEM) confirmed the presence of nanofibers in both the diluent and RG PA samples, suggesting that the incorporation of the bioactive epitope did not negatively impact the self-assembly of these supramolecular polymers (Figure 1C,D). In both the diluent PA (Figure 1C) and the RG PA (Figure 1D), elongated nanofibers were observed. To further probe the internal ordering within the supramolecular polymers, circular dichroism (CD) was performed. PA monomers are held together through strong intermolecular interactions, primarily beta-sheet hydrogen bonding between hydrophobic amino acids adjacent to the hydrophobic tail of the monomer. Both the diluent PA and RG PA-coassembled nanofibers exhibited the characteristic beta-sheet minima at 218 nm and maxima at 195 nm (Figure 1E).^50^ Although the same features are observed in the coassembled RG PA nanofiber structure, suggesting that the secondary structure was maintained through the incorporation of a bioactive epitope, there are noticeable differences in the intensity of the maxima and a shift of the minima. We hypothesize that the observed broadening of the CD minima is likely a result of a more dynamic structure due to the incorporation of the REGRT bioactive peptide epitope affecting the close packing that is possible between monomers when compared to the diluent PA nanofibers. The resulting structure likely has reduced heterogeneity in hydrogen bonding, which manifests as widened minima with a slight blue shift, indicative of reduced beta-twists, as observed previously.^51,52^ Furthermore, an increase in PA monomer charge has previously been shown to increase molecular dynamics of these nanofiber systems, especially with zwitterionic peptide sequences, similar to our REGRT bioactive PA sequence.^53,54^

### Scaffold Formation and Characterization

PA nanofibers can be ionically cross-linked to tune both viscosity and stiffness of the hydrogel produced, an important consideration for topical applications. The gamma-carboxyl groups of glutamic acid on the PA monomers can chelate divalent cations, forming an ionically cross-linked supramolecular polymer matrix.^33,34,55^ The viscosity of the PAs following cross-linking was investigated using a steady-rate sweep test on a cone–plate rheometer, where the diluent and RG PA nanofibers displayed similar rheological profiles and viscosities (Figure 2A). Both are shear-thinning, which is to be expected from supramolecular polymers; the applied shear at all rates was sufficient to disrupt entanglements between nanofibers and align chains.^56^ A strain sweep was performed at a fixed frequency of 1 Hz to elucidate the linear viscoelastic region (LVR) for each material, providing insight into the mechanical stability of the hydrogels (Figure 2B). The diluent PA resulted in a stiffer hydrogel, as indicated by a higher elastic modulus (*G*′) and a truncated LVR, in comparison to the RG PA nanofibers. The RG PA exhibited an extended LVR, while maintaining a lower *G*′, which is indicative of the materials’ ability to maintain its structural integrity over wider strains, because of the reduced stiffness of the network. Functionally, this less stiff material requires higher strain to exhibit liquid-like deformation. However, in applications such as syringe delivery or topical application, this difference is negligible, as both materials deform under sufficiently high strain and subsequently reform.

**Figure 2:**
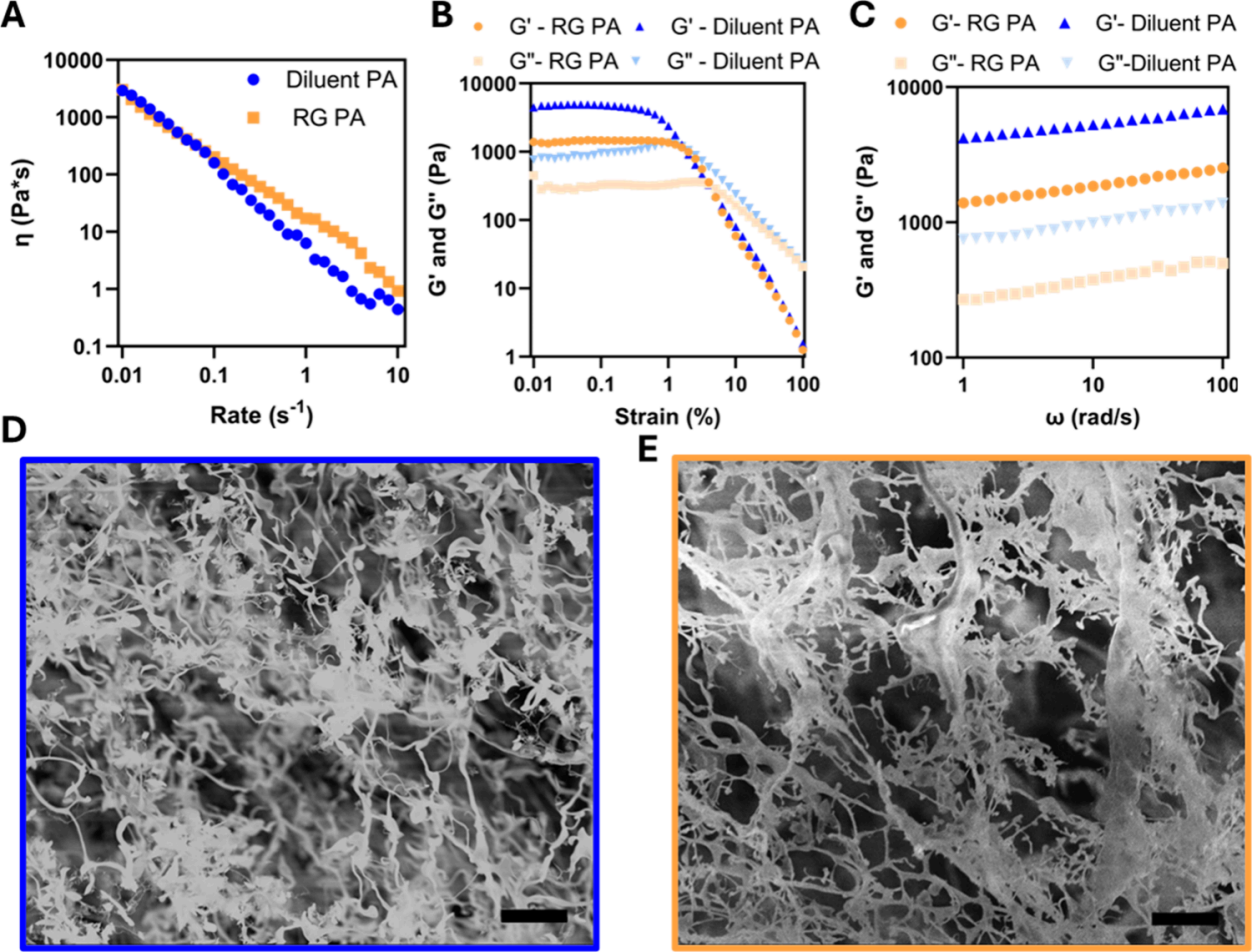
Mechanical properties and morphology of the bioactive PA nanofiber scaffolds. (A) Shear rate sweep from 0.01 to 10 s^–1^ to display viscosity differences between ionically cross-linked PA nanofiber samples. (B) Dynamic strain sweep from 0.01 to 100% to determine the linear viscoelastic regime (LVR). (C) Frequency sweep at fixed strain to determine elastic modulus (*G′*). Scanning electron microscopy (SEM) images highlighting topography of (D) the diluent PA nanofibers and (E) the RG PA nanofibers. SEM scale bars are 10 μm.

Finally, a frequency sweep was performed at 0.1% strain, within the LVR of both materials, where *G*′ was expected to dominate *G*″ as is standard for cross-linked hydrogels.^57,58^ The elastic modulus, which is indicative of stiffness of the material, increased with increasing frequency. As such, the storage modulus dominates the loss modulus and a greater resistance to deformation was observed. This trend was observed for both hydrogels, with the RG PA nanofibers having an elastic modulus an order of magnitude lower than that of the diluent PA nanofibers (Figure 2C). The incorporation of the bioactive REGRT epitope and flexible linker modified fibril packing and entanglement, as observed by TEM and CD analysis (Figure 1C–E), thereby altering the stiffness of the gel following calcium chelation and leading to the formation of less rigid networks. Practically, both gels will resist rapid cyclic forces, appearing stiffer under high applied frequency, making them strong candidates for tissue engineering scaffolds. To better understand the topography of the PA nanofiber scaffolds and scaffold porosity, scanning electron microscopy (SEM) was utilized. Both PA nanofiber scaffolds appeared to form microporous architectures with no differences observed from the inclusion of the bioactive REGRT peptide sequence in the RG PA nanofibers (Figure 2D,E).

To assess cellular cytotoxicity and potential for cell incorporation within the scaffolds, PA nanofibers were evaluated in the presence of human embryonic kidney cells (HEK293). At lower PA nanofiber concentrations in solution, cytotoxicity was assessed using a lactate dehydrogenase (LDH) assay, where no significant increase in cell cytotoxicity was observed following 24 h of incubation with either of the PA nanofibers (Figure S4). Importantly, the addition of the cationic bioactive REGRT peptide epitope, when tethered to the hydrophobic tail of the PAs, did not induce additional cytotoxicity when compared to the diluent PA or no treatment controls. The cytotoxicity of PA nanofiber scaffolds was assessed by ionically cross-linking the nanofibers, seeding HEK293 cells onto the scaffolds, and performing subsequent LDH assessment (Figure 3A). A slightly decreased cytotoxicity compared to the no treatment control was observed from the PA nanofiber hydrogels, which we hypothesize is due to the cells being distributed throughout the scaffold, where the hydrogel restricts the release of LDH into the supernatant.

**Figure 3:**
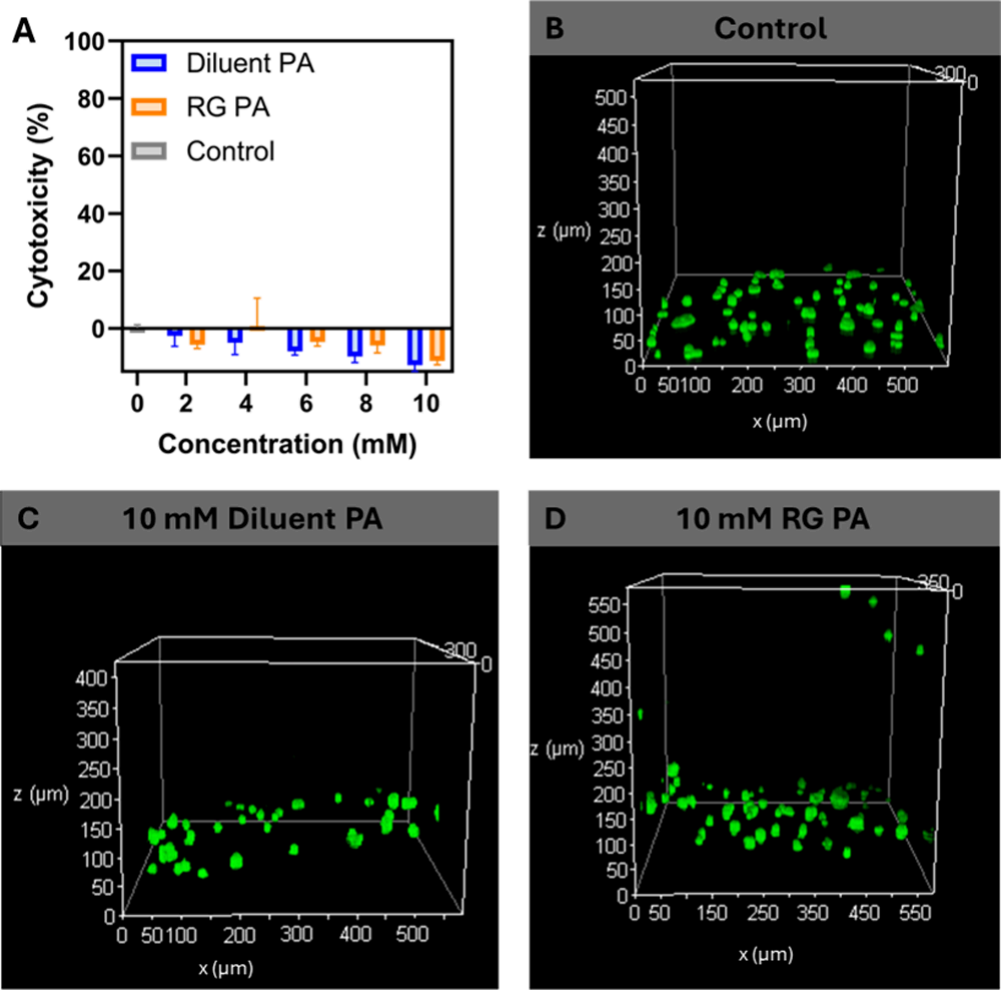
Cytotoxicity assessment of the bioactive PA nanofiber scaffolds. (A) Lactate dehydrogenase (LDH) assay on ionically cross-linked PA nanofiber scaffolds. Confocal microscopy Z-stack reconstructions of live cells stained with Calcein-AM following 24 h of incubation with (B) no treatment control, (C) cells migrating through the diluent PA scaffold (10 mM), and (D) cells migrating through the bioactive RG PA scaffold (10 mM).

To further confirm cell viability, Calcein-AM staining was performed to visualize live cells, where no differences in cell viability were observed by confocal microscopy. All cells were seeded on top of the scaffolds and monitored after 24 h of incubation, where it was observed that the cells effectively migrate into and through the matrix at all concentrations assessed (Figure 3B–H). Taken together these observations demonstrate that the PA nanofibers are noncytotoxic and are suitable as scaffolds to allow cell infiltration and migration throughout, important properties for supporting wound healing following a severe burn injury.

Following characterization of the PA nanofibers, their applicability to severe burn injury was evaluated using an *in vivo* murine burn injury model. A clinically relevant mouse model of deep dermal burn injury sustained from thermal contact was employed to assess the ability of the PA nanofibers to impact wound healing kinetics and final wound closure (Figure 4).^59^ The model is described in detail in the Supporting Information; however, briefly, full-thickness wounds were created in mice using a heated billet to generate six 1 cm^2^ wounds per mouse, resulting in an approximately 20% total body surface area (TBSA) burn, consistent with previously established protocols.^60^ Full thickness was determined by the operator performing the procedure to ensure that the burn was through the epidermis and dermis. The size of the wound was important in this study, as a 20% TBSA burn is sufficient to induce burn shock. Burn shock is a compounding factor in treatment, characterized by an acute systemic response marked by increased capillary permeability, elevated hydrostatic pressure, leakage of protein and fluid from the intravascular to the interstitial space, reduced cardiac output, and hypovolemia requiring fluid resuscitation.^10,61,62^ The following day, the mice were anesthetized using isoflurane, and the formed eschar was surgically removed using forceps and a scalpel blade to produce a vascularized and debrided wound bed, a protocol highly analogous to current clinical practice. The ionically cross-linked PA nanofibers were topically applied using a syringe, yielding a hydrogel scaffold on the wound bed, which was several millimeters thick. This hydrogel was then covered with a transparent dressing and secured with elastic bandaging.

**Figure 4:**
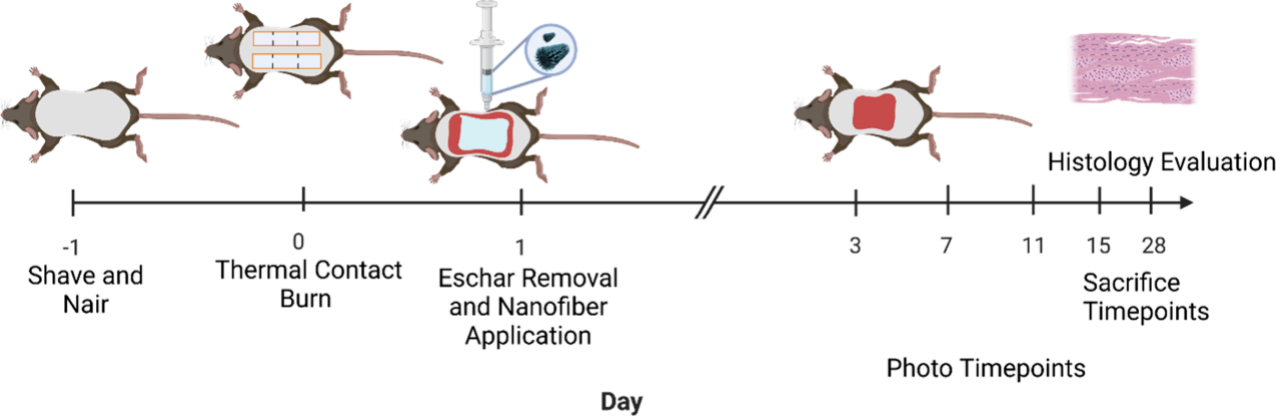
Schematic of deep dermal burn injury murine model utilized in this study.

The burn target of 20% TBSA in mice created heightened variability with healing and challenges in sustained viability. To mimic the true challenges of burn patient populations, this model experienced a moderate level of variability in burn wounds from the thermal contact, size of wound following eschar removal, and mouse healing. The use of such a model provides insight into challenges in burn wound healing, where no two patients or burns would heal alike. Although excision models and smaller thermal wounds promote understanding of skin healing mechanisms and ability of treatments to impact isolated and controlled wounds, the use of a more aggressive model, mimetic of large burn insults (i.e., 20% TBSA) greatly informs the clinical translatability of potential burn injury interventions.

Wound healing kinetics were tracked using digital planimetry where the wound outline was traced to determine wound size over time using ImageJ software.^63^ At early time points, day 3 and day 7, the bioactive RG PA nanofibers demonstrated significantly faster wound closure when compared to the Ringer’s Lactate Solution control-treated mice (Figure 5A,B). The REGRT peptide sequence has been shown to activate the ERK pathway and reduce inflammation in diabetic wound models, which generally benefits early-stage wound healing.^40,41,64^ Enhanced early wound healing suggests that the bioactivity was maintained while tethered to the PA backbone, providing an ECM mimetic structure for cells to migrate into, while also performing as a therapeutic, providing inflammation relief and enhancing cellular migration into the scaffold (Figure 5A). This accelerated healing was achieved with only 20 mol % of the REGRT bioactive epitope in the nanofiber, highlighting the potential for increased loading to further enhance bioactivity. However, such modifications are not straightforward, as higher epitope concentrations may also affect scaffold stiffness and bioactivity in a nonlinear and potentially adverse manner. While we attribute the observed improvements in wound healing to ERK pathway activation by the REGRT motif, we acknowledge that this mechanism has not been directly validated within the scope of this study. By the 7-day time point, the diluent PA nanofibers demonstrated significant improvements in wound closure compared to the Ringer’s control-treated burns (Figure 5B). This aligns well with the proliferative phase of wound healing, where the benefit of having an ECM mimetic scaffold to support cell infiltration has been observed previously.^65,66^

**Figure 5:**
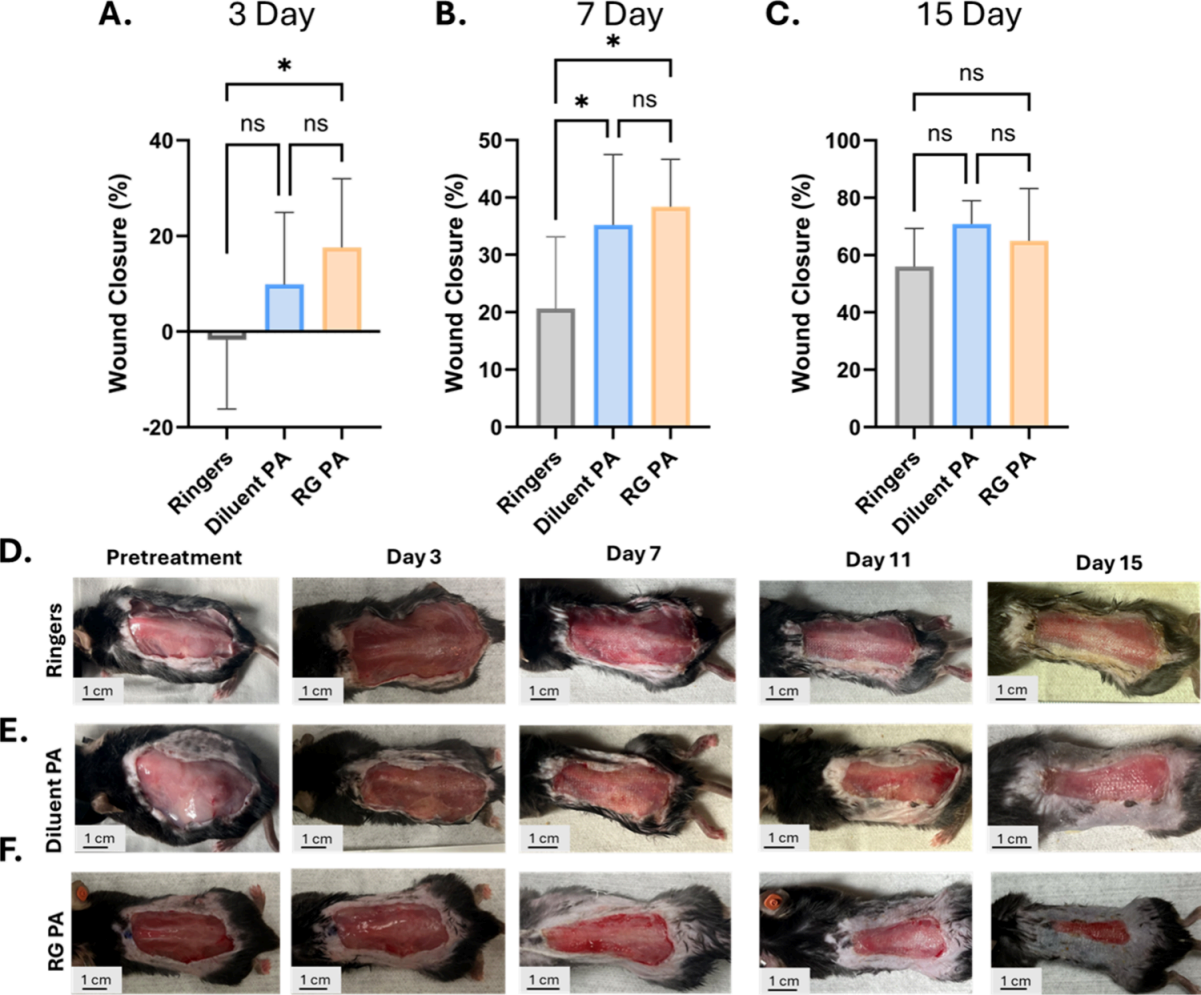
Evaluation of murine burn injury model wound healing kinetics. (A) Three-day wound closure quantification (*p = 0.017), (B) 7-day wound closure quantification (*p*-value Ringers compared to diluent PA *p = 0.0382, *p*-value Ringers to RG PA *p = 0.017), and (C) 15-day wound closure quantification. Statistical significance was determined in GraphPad Prism Statistical Software with a one-way ANOVA and *post hoc* Tukey test for difference in means comparison. Minimum *n* = 7 for each time point evaluated, *p < 0.05. Representative images of wound healing kinetics for (D) Ringers control, (E) diluent PA nanofiber treatment, and (F) bioactive RG PA nanofiber treatment.

By day 15, the healing of all treatments begins to converge as remodeling and maturation occur, and final contracture of the wound dominates (Figure 5C). Representative images of the wound healing time course for each treatment group demonstrate that the inclusion of PA nanofibers promotes deep dermal wound healing, especially at early time points (Figure 5D–F). This accelerated wound closure has the potential to reduce the risk of infection, minimize the opportunity for final scarring, and improve the overall patient recovery.^24^ The severity of the model and variability of wound healing within the mice made this a strong representation of severe, clinically relevant burn injury. The acceleration of wound closure observed by the bioactive RG PA nanofibers provides an exciting basis for improving treatments of deep dermal wounds and other chronic wounds where the pathophysiology is influenced by increased inflammation.

At the time of sacrifice, skin samples were collected for histology analysis by hematoxylin and eosin (H&E) and collagen assessment with Masson’s Trichrome staining. Hematoxylin and eosin staining of excised wound tissue at 15 days postinjury revealed marked differences in tissue architecture across the treatment groups (Figure 6A–C). In the Ringer’s-treated group, re-epithelialization was minimal, with sparse epidermal coverage and poorly organized dermal structure, indicating limited wound resolution (Figure 6A). The underlying tissue exhibited low cellularity and incomplete granulation tissue formation, consistent with a stalled or delayed healing response. In contrast, wounds treated with the diluent PA scaffold demonstrated moderate re-epithelialization (Figure 6B), with a thicker and more continuous epidermal layer compared to the Ringer’s only control. The dermis showed increased cellular infiltration and early granulation tissue formation, suggesting that the scaffold provided a degree of structural support conducive to tissue repair, despite lacking bioactive functionality. The most advanced healing response was observed in the RG PA-treated group, which exhibited a well-defined, stratified epidermis spanning the wound site (Figure 6C). The underlying dermis was densely cellular and highly organized, with extensive granulation tissue indicative of active-matrix remodeling and tissue regeneration. These results suggest that the bioactive RG PA scaffold enhances both epidermal regeneration and dermal repair, supporting its potential therapeutic benefit in promoting more complete wound healing following severe burn injury. At 28 days, all groups exhibited complete re-epithelialization, with the Ringer’s control group showing a well-organized epidermis and mature dermis, while diluent PA and RG PA display thicker epidermal layers and increased dermal cellularity by comparison (28-day comparison, Figure 6A–C). Importantly, the biocompatible PA scaffolds did not display any inflammatory response at 15 or 28 days post-treatment, as assessed by H&E staining. This is consistent with comparable PA nanofibers, which have shown excellent biocompatibility and persistence of the PA nanofibers at 1 month following implantation in a dorsal skinfold chamber model.^67^

**Figure 6:**
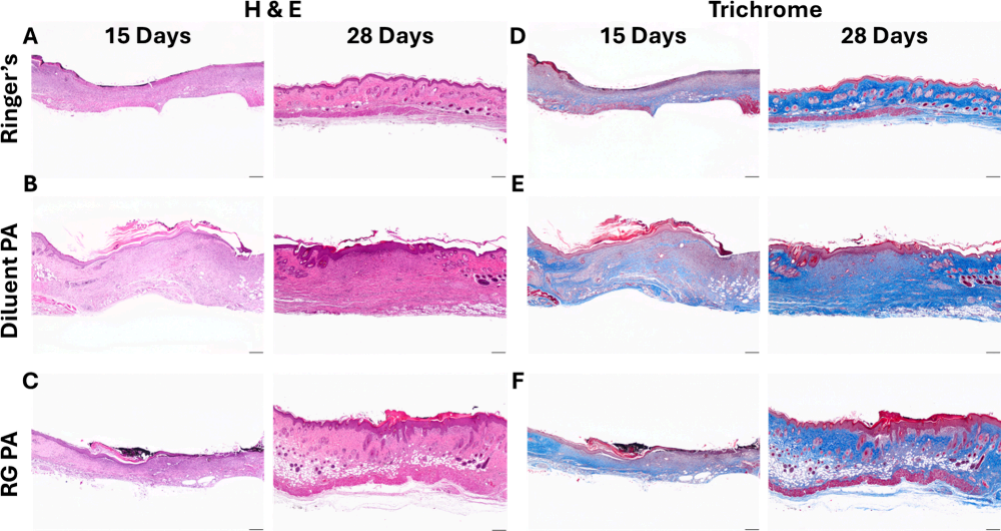
Histological evaluation of healed wounds at the 15- and 28-day time points. Representative hematoxylin and eosin (H&E) staining at the 15-day and 28-day time points for (A) Ringer’s Control treatment, (B) diluent PA nanofibers, and (C) bioactive RG PA nanofibers. Masson’s trichrome staining at the 15-day and 28-day time points for (D) Ringer’s Control treatment, (E) diluent PA nanofibers, and (F) bioactive RG PA nanofibers. All scale bars are 200 μm.

Masson’s Trichrome staining was used to evaluate ECM deposition and collagen organization in wound tissues at 15 and 28 days postinjury (Figure 6D–F). At day 15, wounds treated with Ringer’s solution exhibited limited collagen deposition, with diffuse and discontinuous blue staining within the wound bed (Figure 6D). The dermal layer appeared thin and poorly organized, with minimal matrix remodeling evident. In contrast, wounds treated with the diluent PA scaffold showed enhanced collagen deposition, evidenced by increased and more uniform blue staining within the granulation tissue, though the matrix remained loosely structured (Figure 6E). Notably, the RG PA nanofiber-treated group demonstrated the most extensive collagen presence at this time point, with dense, blue-stained collagen distributed throughout the wound site and early signs of dermal matrix organization, suggesting that the bioactive RG PA scaffold promoted early ECM remodeling (Figure 6F). By day 28, all treatment groups demonstrated more extensive collagen deposition relative to day 15. In the Ringer’s control group, collagen was more evenly distributed, and dermal architecture appeared more mature, though overall collagen density remained lower than in PA nanofiber-treated groups. The diluent PA group exhibited further maturation of the matrix, with deeper and more organized collagen layers and reduced cellularity, indicating ongoing remodeling. The RG PA nanofiber treatment demonstrated collagen remodeling that extended deeper into the wound bed, and the granulation tissue had begun to resolve, reflecting a transition toward regenerative tissue architecture.

## Conclusions

Burn injuries remain among the most devastating medical conditions faced in clinical practice, leading to significant morbidity, mortality, and long-term scarring. Despite advancements in treatment, current approaches for severe burns are hindered by challenges such as poor graft integration, scarring, infection risk, and limited availability of donor tissue. Herein, we demonstrated that PA nanofiber scaffolds, particularly those functionalized with the bioactive REGRT sequence, offer a promising therapeutic strategy for enhancing wound healing in deep dermal burn injuries. Our findings indicate that the bioactive RG PA significantly accelerates wound closure at early time points, likely due to its ability to modulate inflammation, promote keratinocyte migration, and provide a bioactive ECM mimetic scaffold for cellular infiltration. The nanofiber structure of the PA scaffolds, combined with their tunable mechanical properties, supports the regeneration of skin tissue, while reducing the need for secondary interventions, such as revision surgeries as is required with other synthetic scaffolds available. This was demonstrated with a clinically relevant murine model, utilizing a large thermal burn injury (20% TBSA) with eschar removal, contributing further to the translational potential of this approach. The bioactive RG PA scaffolds demonstrated excellent mechanical properties and cytocompatibility, are bioactive, and are biodegradable consisting purely of lipids and amino acids. These results underscore the potential of PA-based scaffolds to support the treatment of severe burns and advance regenerative medicine strategies aimed at enhancing wound closure, restoring functional skin, and reducing the risks of infection and long-term scarring.

## Supplementary Material



## Notes

LC-MS, CAC determination, cytotoxicity of ungelled PAs, and confocal microscopy (PDF)
