# Supplementary material for: Bioactive Supramolecular Polymers for Skin Regeneration Following Burn Injury
Source: Biomacromolecules. 2025 Jul 16;26(8):5471–82. doi: 10.1021/acs.biomac.5c01107 (PMC12344711; doi:10.1021/acs.biomac.5c01107)
Supplement: Supplementary file 1 [file bm5c01107_si_001.pdf]

Supporting Information

Bioactive Supramolecular Polymers for Skin Regeneration Following Burn Injury

Penelope E. Jankoski,[1] Abdul-Razak Masoud,[2] Jenna Dennis,[2] Sophia Trinh,[2] Loria R. DiMartino,[1] Jessica Shrestha,[1] Luis Marrero,[2] Jeffery Hobden,[2] Jeffrey Carter,[2,3] Jonathan Schoen,[2,3] Herbert Phelan,[2,3] Alison A. Smith,[2,3]* and Tristan D. Clemons.[1,4*]

1 School of Polymer Science and Engineering, University of Southern Mississippi, Hattiesburg, MS, 39406, USA.

2Louisiana State University Health Sciences Center, New Orleans, LA 70112, USA

3 University Medical Center, New Orleans, LA 70112, USA

4 Center for Molecular and Cellular Biosciences, University of Southern Mississippi, Hattiesburg, Mississippi 39406, USA.

*Correspondence should be addressed to T.D.C. (email: Tristan.clemons@usm.edu) and A.S. (email: asmi60@lsuhsc.edu).

Supporting Information

Table of Contents

1. **Materials and Methods** 3
1.1 Materials 3
1.2 Peptide Amphiphile Synthesis 3
1.2.1 Diluent PA Synthesis 3
1.2.2 REGRT PA Synthesis 4
1.3 Peptide Amphiphile Nanofiber Preparation 4
1.4 Peptide Amphiphile Nanofiber Preparation for Animal Studies 4
2. Material Characterization 4
2.1 Liquid Chromatography Mass Spectrometry (LC-MS) 4
2.2 Nile Red Assay 4
2.3 Circular Dichroism (CD) 5
2.4 Transmission Electron Microscopy (TEM) 5
2.5 Scanning Electron Microscopy (SEM) 5
2.6 Rheology 5
2.7 Cell Work 6
2.7.1 Cell Culture Maintenance 6
2.7.2 Cytotoxicity 6
2.7.3 Live Well Confocal Imaging 6
2.8 In Vivo Studies 7
2.8.1 Burn Wound Model 7
2.8.2 Kinetics of Healing 7
2.8.3 Histological Evaluation 8
3. Supplemental Figures 8
Figure S1. LC-MS analysis of Control PA 8
Figure S2. LC-MS analysis of RG PA. 9
Figure S3. Critical aggregate concentration determination by Nile Red assay 10
Figure S4. PA nanofiber cytotoxicity assessment. 10
Figure S5. Confocal microscopy live cell imaging within PA nanofiber scaffolds at varying concentrations of the PA nanofibers. 11
4. References 11

1. Materials and Methods

1.1 Materials

Rink amide Polystyrene Resin, Fmoc protected amino acids, and ethyl cyanoglyoxlate-2oxime (Oxyma) were purchased from CEM peptides. Dichloromethane (DCM), diethyl ether, trifluoroacetic acid (TFA), N-dimethylformamide (DMF), acetonitrile, diisopropylcarbodiimide (DIC), triisopropylsilane (TIS), ethane-1,2-dithiol (EDT), Uranyl Acetate and all other solvents were purchased from ThermoFisher Scientific (USA) or Sigma Aldrich Corporation (USA) at the highest purity. Dulbecco's modified eagle medium (DMEM), heat inactivated fetal bovine serum (FBS), and penicillin-streptomycin were all purchased from ThermoFisher, sterile, and tissue treated 96-well plates were obtained from CellTreat (USA). The CyQUANT Lactate Dehydrogenase (LDH) Assay was purchased from ThermoFisher (USA). Hexafluoroisopropanol (HFIP) was obtained from AA blocks and Nile Red was purchased from ApexBio. Isoflurane USP was purchased from Covetrus (Batch # G48D23A) (USA). Meloxicam ER (Lot # 222-05468474) and Buprenorphine HCl in polymer (Lot # 222-05509534) were purchased from Wedgewood Pharmacy (USA). Mason's Trichrome staining kit was purchased from StatLab (SKU #: KTMTR2LT) (USA).

1.2 Peptide Amphiphile Synthesis

Peptide Amphiphiles were synthesized on a Liberty Blue 2.0 automated peptide synthesizer (CEM) through standard 9-fluorenyl methoxycarbonyl (Fmoc)- based solid phase peptide synthesis. Peptide synthesis was performed at 0.25 mmol scale using Rink Amide Polystyrene Resin (0.3mmol/g loading, 100-200 mesh). Deprotection of Fmoc protecting groups was carried out using 20 v/v% piperidine in DMF. Each amino acid addition was carried out using Fmoc-protected amino acids (0.2 M), DIC (1M), and Oxyma (1 M) in DMF. After the final Fmoc deprotection, the resin beads were washed 3x using DCM. The peptide then underwent global deprotection and cleavage from the resin beads through gentle shaking in TFA/TIS/H_2O/EDT (95: 2.5:2.5:2.5) cleavage cocktail for 3 hours at room temperature. Peptide amphiphiles were then precipitated in cold diethyl ether and collected via centrifugation. The peptide pellet was then resuspended in diethyl ether and chilled for four hours. It was recentrifuged and the diethyl ether supernatant was decanted from the peptide pellet, which in turn was allowed to air dry. Crude peptides were purified on a Prodigy preparative reverse-phase HPLC (CEM) with a water/acetonitrile gradient (containing 0.1% NH_4OH). The mass and identity of the eluting fractions containing the desired peptides were confirmed using electrospray ionization (ESI)- mass spectrometry (MS) on a Thermo Scientific Orbitrap Exploris™ 240. Purity was confirmed using liquid-chromatography mass-spectrometry, with a demonstrated purity of greater than 95%. Initial targets for this work were synthesized at the NSF-supported BioPACIFIC MIP facilities following similar protocols as above utilizing a Gyros Protein Technologies Symphony® X Peptide Synthesizer.

1.2.1 Diluent PA Synthesis

The following peptide sequence $C_{16}V_3A_3E_3$ was synthesized on Rink amide MBHA resin making use of the CEM Liberty microwave-assisted peptide synthesizer and protocols described above in section 1.2.

1.2.2 REGRT PA Synthesis

The following peptide sequence $C_{16}V_3A_3E_3G_4$REGRT was synthesized on Rink amide MBHA resin making use of the CEM Liberty microwave-assisted peptide synthesizer and protocols described above in section 1.2.

1.3 Peptide Amphiphile Nanofiber Preparation

The resulting powders were dissolved in 100 mM HCl and lyophilized to neutralize any lingering TFA. The PAs were then weighed out into Eppendorf tubes to be at 10 mM concentration with a working volume of 1 mL. The PAs were then dissolved in milli-Q water and slowly pH adjusted to a pH between 7 and 8 using 1 M NaOH being careful not to overshoot. The PAs were then lyophilized and resuspended in working volume to obtain 10 mM stock solutions. Diluent PA was stored at – 20 °C until use. To prepare the 20% bioactive PA, 200 µL of 10 mM REGRT PA and 800 µL of 10 mM diluent PA were mixed. The bioactive PA was then lyophilized. The dry powders were dissolved in HFIP and left to evaporate overnight and then redissolved in 1 mL of DI water and lyophilized.

1.4 Peptide Amphiphile Nanofiber Preparation for Animal Studies

PAs for application to animals were dissolved in 990 uL of sterile Ringers. These PAs were thermally annealed at 80 °C for 30 minutes and allowed to slow cool back to room temperature. For animal studies, 1 M $CaCl_2$ was prepared in DI water and sterilized using the liquid setting on the autoclave. Following annealing 10 µL of $CaCl_2$ was added to promote gelation through ionic crosslinking. Sterile Ringers was prepared with an equivalent amount of $CaCl_2$ to act as a negative control.

2. Material Characterization

2.1 Liquid Chromatography Mass Spectrometry (LC-MS)

The purity of PA molecules was confirmed using liquid chromatograph-mass spectroscopy (LC-MS), which was performed using an Agilent 1200 system with a Phenomenex Gemini C-18 column (100 × 1.00 mm; 5 µm) for basic conditions. The mass detector (MS) was an Agilent 6520 Q-TOF MS. All gradient methods followed: acetonitrile at 5% for 5 min at 50 µL/min, 5–95% over 25 min at 50 µL/min followed by 95% for 5 min at 50 µL/min. Ammonium hydroxide (0.1% v/v) for basic conditions was added to all solvents. Peaks were detected at λ = 220 nm.

2.2 Nile Red Assay

Stock solutions of Nile Red were prepared at 10 mM in DMSO and subsequently diluted with deionized (DI) water to achieve a final working concentration of 100 µM. PA samples were diluted in DI water to produce a concentration range of 0 to 500 µM. In a 96-well plate, 90 µL of PA solution and 10 µL of Nile Red solution were added to each well, mixed thoroughly, and incubated

at room temperature for 3 hours, with intermittent tapping to promote incorporation. Each condition was performed in triplicate. After incubation, samples were analyzed using a Biotek Synergy H1 Microplate Reader (Agilent), with excitation set to 550 nm and emission measured in 2 nm increments from 580 to 720 nm. The mean maximum relative fluorescence units (RFU) were plotted against the logarithm of the concentration, and the critical aggregation concentration (CAC) was determined as the intersection point of the curves corresponding to the absence and presence of fluorescence.

2.3 Circular Dichroism (CD)

PAs were prepared as described above and diluted to 100 – 500 μMin milli-q water. CD spectra was recorded in a 1 mm pathlength cuvette on a J-815 (Jasco, Easton, MD) spectropolarimeter. Continuous scanning mode was used with a scanning speed of 100 nm per minute over a measurement range of 190 – 300 nm. The high-tension voltage (HT) was also monitored to ensure that the measurement was not saturated. Three measurements were obtained, and the buffer sample was run as a background that was subtracted.

2.4 Transmission Electron Microscopy (TEM)

200-mesh copper grids (Ted Pella) were used as purchased. PA solution was diluted to 0.5 mM and 5 μL was dropped on the grid and left to sit for 5 minutes. Excess solution was wicked away and a drop of uranyl acetate was added as a stain to sit for 2 minutes. Excess solution was wicked away and 5 μL of deionized water was added and left to sit for 5 minutes. The solution was wicked away and grids were left to air dry prior to visualization using a JEOL JEM120i Transmission Electron Microscope.

2.5 Scanning Electron Microscopy (SEM)

Stainless steel stubs were prepped with adhesive carbon black conductive tape. PA solutions were diluted to 2 mM and applied dropwise. Samples were then left to sit for 5 minutes and lyophilized using a Benchtop Pro Lyophilizer (SP Scientific). Samples remained under vacuum until they were run on SEM to prevent absorption of water as they are hygroscopic. Samples were run using a Zeiss Sigma VP field-emission SEM in an N_2 environment.

2.6 Rheology

Rheological measurements were conducted using a strain-controlled ARES rheometer (TA Instruments) fitted with 25 mm cone and plate steel geometry, maintaining a gap height of 0.3 ± 0.05 mm. To establish the viscosity profile of the material, a steady rate sweep test was performed across a shear rate range of 0.01 to 10 s^{-1}. 450 μL of PA nanofibers (10mM in DI water prepared from lyophilized stocks as described in section 1.3) was placed on the steel plate, followed by an equal volume of 10 mM $CaCl_2$ solution pipetted on top. The samples were allowed to gel for 15 minutes, after which 450 μL of the gelling solution was carefully removed, and the test was performed. Measurements about the storage and loss modulus of the samples were made using dynamic motor mode, with crosslinked 10 mM PA samples as described above. To determine the linear viscoelastic regime, a dynamic strain sweep was performed, holding the frequency constant at 1 Hz. The PAs were investigated using a dynamic strain sweep from 0.01 – 100% Strain. Then a frequency sweep from 1 – 100 rad/s was performed at a fixed strain of 1%, to determine the material response within the LVR where solid like behavior dominates.

2.7 Cell Work

2.7.1 Cell Culture Maintenance

Human embryonic kidney cells (HEK 293) were maintained in Dulbecco's modified eagle medium, supplemented with 10 % fetal bovine serum (FBS) and 0.1% penicillin-streptomycin. Cells were cultured at 37° C, 5% CO_2 in tissue treated flasks and used at confluence of ~ 80%.

2.7.2 Cytotoxicity

HEK293 cells were seeded in a 96 well plate (1 x 10^5 cells/mL, 100 μL volume per well). Seeded cells were incubated at 37 °C and 5% CO_2 overnight to allow cells to adhere. Following adherence, PA solution was added to the media to incubate, and each concentration was performed in triplicate. Nuclease free water was used as a spontaneous control, and Triton X-100 was used as a positive control for 100 % cytotoxicity (i.e. complete LDH release). Plates were incubated for 24 hours at 37 °C and 5% CO_2, before collecting media to assess LDH release with the CyQUANT LDH assay following the manufacturers protocols. A microplate reader was used to assess the absorbance at 490 nm with a reference wavelength of 690 nm. % Cytotoxicity was calculated using equations 1 and 2 below.

$$(1)\ LDH\ Activity = A_{sample,490} - A_{sample,680}$$

$$(2)\ \%\ Cytotoxicity\ \left(\frac{LDH\ Activity_{sample} - LDH\ Activity_{spontaneous}}{LDH\ Activity_{Max\ Lysis} - LDH\ Activity_{spontaneous}}\right) * 100$$

Above 2 mM there are significant challenges from the peptide solution being gelled by ions in DMEM, producing nonhomogeneous distributions of PA and cells. In order to obtain LDH data for these higher concentrations, and of the gelled systems as a whole, PAs were gelled within the well by adding 50 μL of sample and 50 μL of 10 mM $CaCl_2$ solution. PAs were left to gel for 15 minutes, 50 uL of excess liquid was pipetted off leaving only gelled PA behind. Cells were then gently plated on top of the gelled layer at 1 x 10^5 cells/mL, 100 μL volume per well and left to incubate overnight. LDH was then performed as described above using supernatant from each well.

2.7.3 Live Well Confocal Imaging

To support the LDH assay results, and visualize cells within the gels, samples were prepared as described in 2.7.2 for gelled cytotoxicity. Cells were plated in a glass bottom 96 well plate (# 1.5 glass) and after 24 hours of coincubation, 70 μL of supernatant was removed from each well and 80 μL of Live stain was added. In this study, ThermoFisher Live/Dead Cell Imaging Kit was used, however, BOBO-3 the dead cell stain complexes with PAs, making visualization of dead cells impossible. Cells were incubated at room temperature for 15 minutes prior to imaging on a Leica STELLARIS STED Super-Resolution Confocal Microscope. Z-stacks were taken at each concentration to visualize cells migrating within the gels.

2.8 In Vivo Studies

2.8.1 Burn Wound Model

Animal experiments were conducted in accordance with protocols approved by the Institutional Animal Care and Use Committee (IACUC) at Louisiana State University Health Sciences Center – New Orleans (Protocol #2403). A total of 36 male C57BL/6J mice (JAX #000664) were procured from Jackson Laboratory (Bar Harbor, ME, USA) for use in this study. Mice were housed under standard conditions (20–26 °C, 12-hour light/dark cycle) and acclimated for one week prior to experimentation. On the day of the experiment, anesthesia was induced using 3% isoflurane in oxygen. The dorsal surface of each mouse was shaved, and analgesia was administered via subcutaneous injection of buprenorphine SR (0.01 mg/kg) and meloxicam SR (5 mg/kg) into the right and left groin regions. Six square full-thickness scald wounds (1 cm × 1 cm) were created on the dorsum using a heated billet applied for 10 seconds. The following day, the formed eschar was surgically removed, and treatment was applied. The burn and subsequent eschar removal generated a wound that was visibly through all layers of the skin, creating the full thickness injury. Mice were randomly assigned to one of three treatment groups: (1) Ringer's solution supplemented with $CaCl_2$, (2) Diluent PA nanofibers, or (3) Bioactive RG PA nanofibers. The Ringer's solution (supplemented with calcium) was a solution of Ringer's lactate which had been enhanced with an addition of $CaCl_2$ to mimic the addition of calcium chloride present to promote ionic gelation within the PAs, resulting in a Ringer's Lactate with 10 mM additional calcium. PAs were prepared as described in section 1.4 resulting in final concentrations of 10 mM PA and 10 mM $CaCl_2$. Approximately 400 µL of treatment were placed on each wound, providing good coverage of the wound area. Wounds were covered with transparent dressings (Tegaderm®) and secured with bandages (Coban). Wound progression was monitored and photographed at defined time points (days 3, 7,11 and 15) following treatment. Pain management was provided as needed based on animal monitoring. At the endpoint mice were euthanized with 5% isoflurane in oxygen over a 40–60-minute period. Wounds along with surrounding skin tissue were harvested and processed for histological analysis.

2.8.2 Kinetics of Healing

Images were taken at Day 0 (burn), Day 1 (eschar removal and treatment), Day 3, Day 7, Day 11, and Day 15 timepoints and analyzed using ImageJ to monitor wound closure over time via digital planimetry. Each picture contained a ruler, which was used to set the scale of pixels to cm^2 to allow for comparison across photographs. Images were analyzed by a single individual to prevent bias and to ensure all images were treated the same. Wound closure was calculated using equation 1 below. Data was then analyzed using GraphPad Prism statistical software (Version 9, Dotmatics, CA, USA) for a one-way analysis of variance (ANOVA) with a post-hoc Tukey test ($\alpha = .05$) for means comparison within each group.

$$\%\,Wound\ Closure = \left(1 - \frac{Area\ of\ Wound_{Day\ n}}{Area\ of\ Wound_{Day\ 1}}\right) * 100$$

2.8.3 Histological Evaluation

Histological evaluation of skin tissue sections was performed using Masson's Trichrome staining, following the manufacturer's protocol. At the time of euthanasia, excised wound tissues were immediately fixed in zinc-buffered formalin, with wound margins marked using India ink. Five (5) µm-thick sections were prepared using a Thermo Scientific HM325 microtome. Sections were subsequently stained with Masson's Trichrome, dehydrated, cleared, and mounted with coverslips. Tissue morphology was examined under a Nikon E300 light microscope equipped with a 4× objective (NA 0.10) and an Olympus DP23 camera. Images of ten fields were taken for each slide. Histological assessment was performed using an established scoring system for murine cutaneous burn wounds and analyses were conducted using Olympus CellSens software.[1]

3. Supplemental Figures

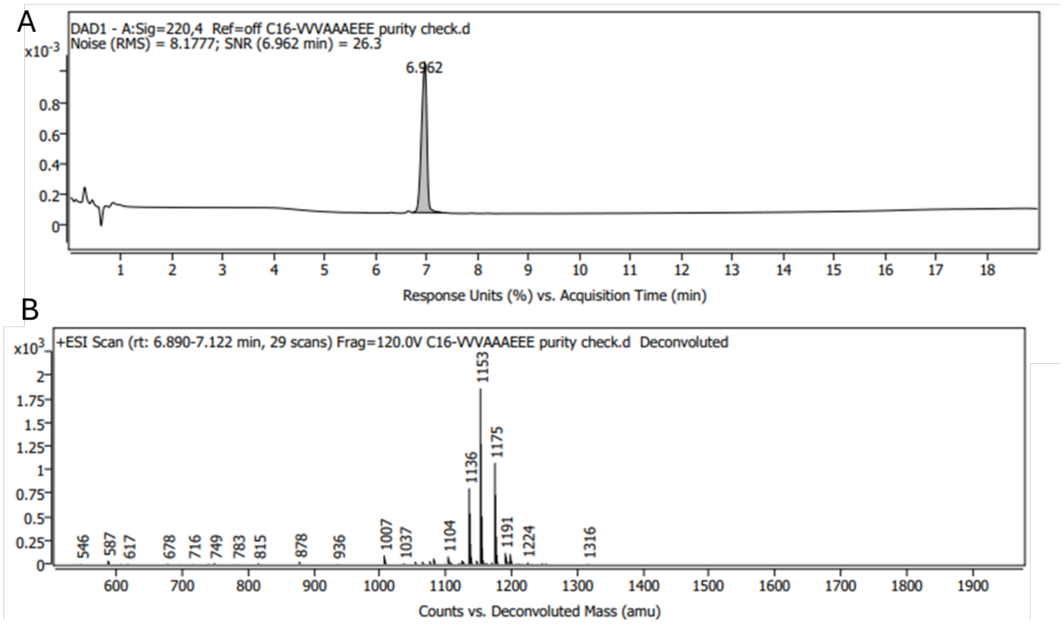

Figure S1. LC-MS analysis of Control PA. a) LC-MS trace of control PA [PA] = 1 mg/mL, loading solvent; H_2O with 0.1% NH_4OH (v/v), eluent; H_2O-CH_3CN gradient containing 0.1% HCOOH (v/v), column; Phenomenex Gemini 5 µm C18 110 Å LC column 150 x 1 mm and ESI-mass spectra corresponding to elution time 6.89 – 7.12 min.

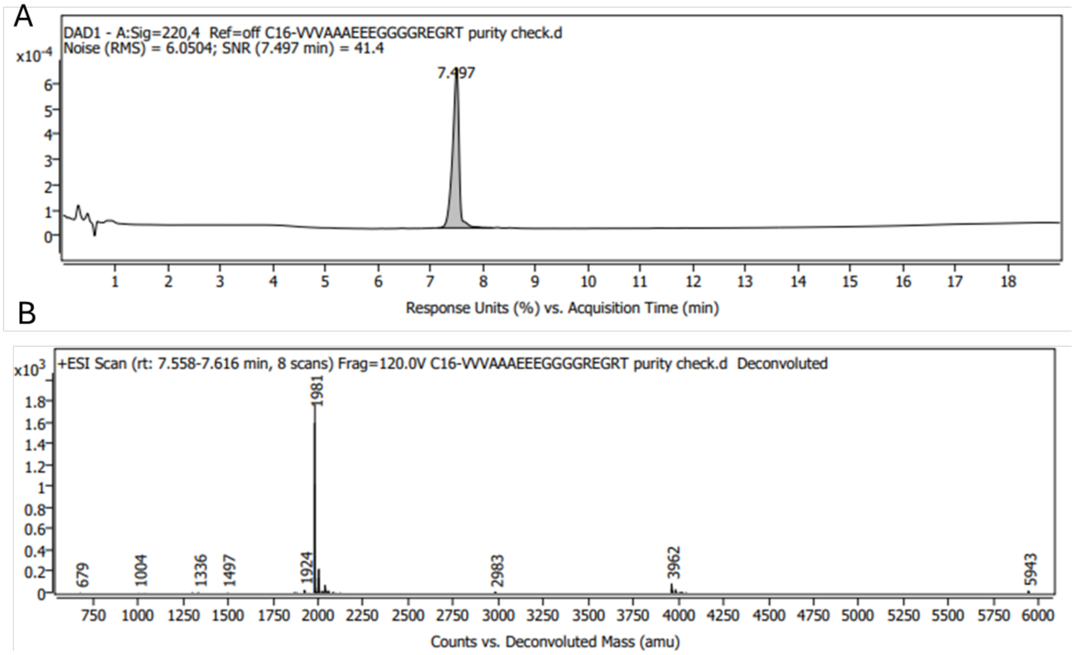

Figure S2. LC-MS analysis of RG PA. a) LC-MS trace of control PA [PA] = 1 mg/mL, loading solvent; H_2O with 0.1% NH_4OH (v/v), eluent; H_2O-CH_3CN gradient containing 0.1% HCOOH (v/v), column; Phenomenex Gemini 5 µm C18 110 Å LC column 150 x 1 mm and ESI-mass spectra corresponding to peak elution time ~ 7.497 min.

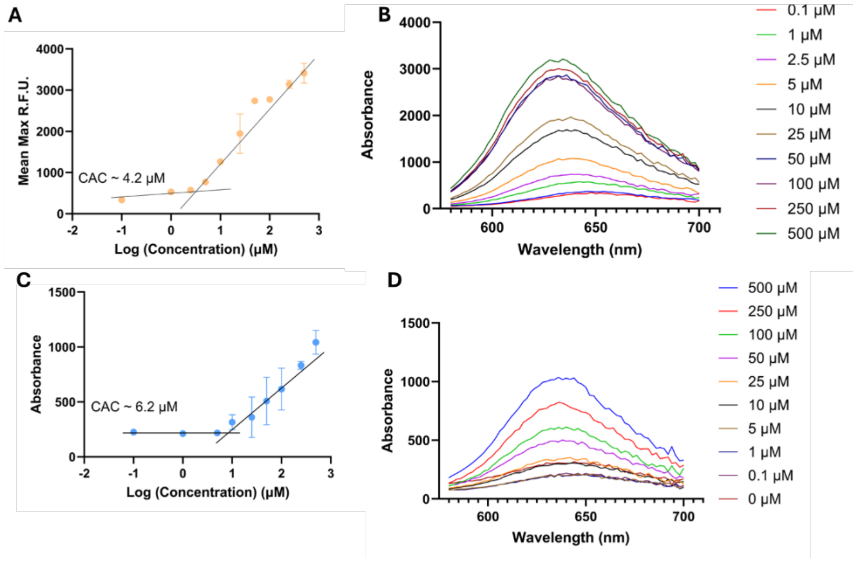

Figure S3. Critical aggregate concentration determination by Nile Red assay. A) Blended 20% RG PA CAC graph and B) spectral scans which were used to calculate the CAC for the 20% RG PA to be 4.2 µM. C) Control PA CAC graph and D) Spectral scans which were used to calculate the CAC for the Control PA to be 6.2 µM.

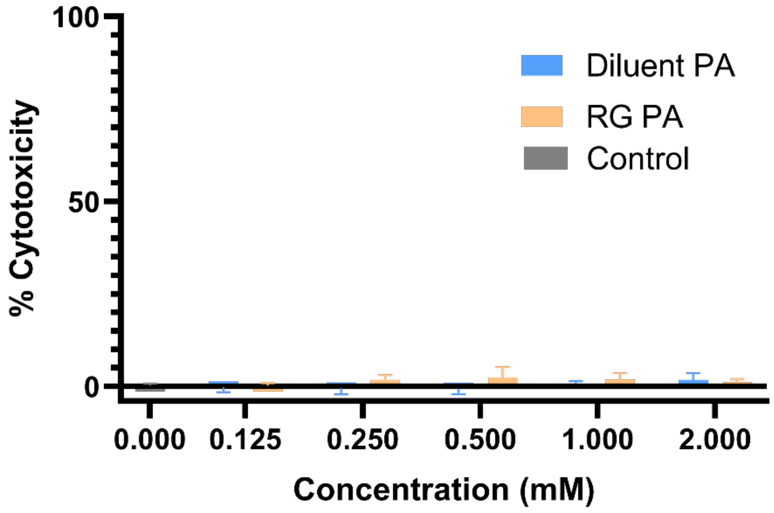

Figure S4. PA nanofiber cytotoxicity assessment. 24-hour cytotoxicity of PAs in HEK 293 cells from LDH Assay highlighting inherent biocompatibility of these materials. Data is reported as mean, s.d. and was analyzed using a one-way ANOVA with post-hoc Tukey test to compare means at α= 0.05 for significance using GraphPad Prism Statistical Software.

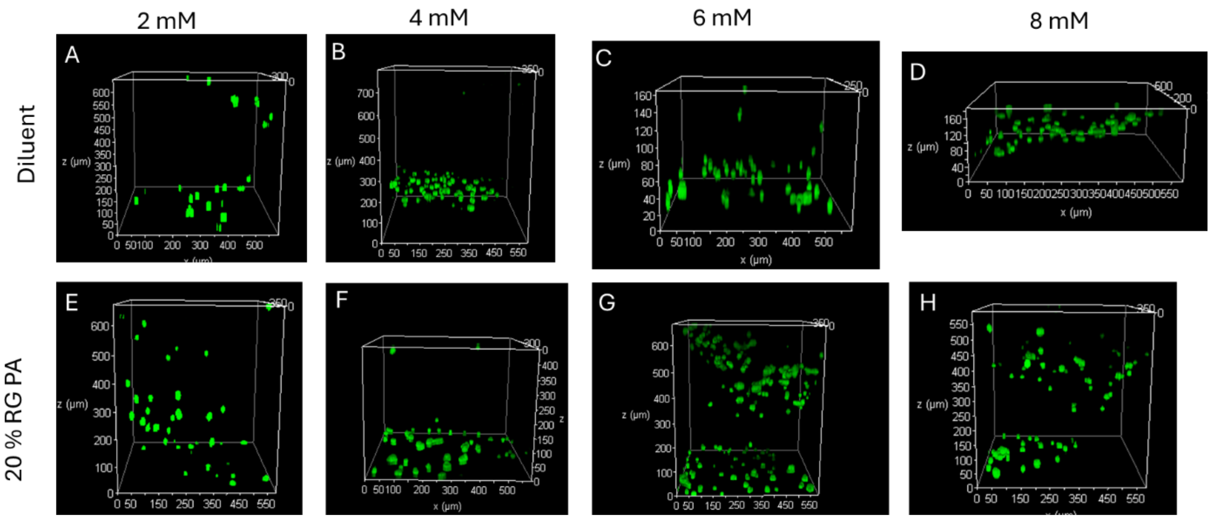

Figure S5. Confocal microscopy live cell imaging within PA nanofiber scaffolds at varying concentrations of the PA nanofibers. Diluent PA at A) 2 mM, B) 4 mM, C) 6 mM, D) 8 mM, and bioactive RG PA at E) 2 mM, F) 4 mM, G) 6 mM, H) 8 mM.

4. References

(1) Van de Vyver, M.; Boodhoo, K.; Frazier, T.; Hamel, K.; Kopcewicz, M.; Levi, B.; Maartens, M.; Machcinska, S.; Nunez, J.; Pagani, C.; et al. Histology Scoring System for Murine Cutaneous Wounds. *Stem Cells Dev* **2021**, *30* (23), 1141-1152. DOI: 10.1089/scd.2021.0124.
